# Functional performance of the upper limb and the most common boxing-related injuries in male boxers: a retrospective, observational, comparative study with non-boxing population

**DOI:** 10.1186/s13102-022-00558-3

**Published:** 2022-09-01

**Authors:** Vasileios Giannatos, Andreas Panagopoulos, Panagiotis Antzoulas, Savvas I. Giakoumakis, John Lakoumentas, Antonis Kouzelis

**Affiliations:** 1 Department of Shoulder and Elbow, Orthopaedic Clinic, Patras University Hospital, Papanikolaou 1, 26504 Rio-Patra, Greece; 2 Special Clinic of Orthopaedic Rehabilitation, Koenig Ludwig, Schwangau, Bavaria Germany; 3 Department of Medical Physics, School of Medicine, Patras University, Patras, Greece

**Keywords:** Boxing, Injury, Upper limb, Hand, Boxer’s knuckle, DASH, PRWE

## Abstract

**Background:**

To investigate the functional status and recording the most common injuries of the upper limb in male Greek boxing squad in comparison to the general population.

**Methods:**

A retrospective injury surveillance study using an electronic questionnaire was performed in 2021. The questionnaire was sent to male members of the Greek Boxing Federation and consisted of three parts. Demographic data, functional status scales, training conditions, hours of training, the location and description of upper limb injuries and whether the injury occurred during competition or training and also whether it was a new or a recurrent one were gathered. The same questionnaire was sent to non-boxer males (military recruits), but without asking them to report any training parameters. Inclusion criteria were age < 35 years-old for all participants and no involvement in martial arts for the control group. Also, all participants (boxers and non-boxers) completed the Patient Rated Wrist Evaluation (PRWE) scale and the Quick Disabilities of Arm, Shoulder and Hand (quick-DASH) score.

**Results:**

The final study cohort was consisted of 62 elite or amateur boxers and 75 non-boxer males, less than 35 years old. The quick-DASH score was found to be significantly lower (better) in boxers in comparison to the general population (15.65 ± 10.25 vs. 12.55 ± 8.62; *p* = 0.020) whereas the PRWE score was similar in both groups (9.25 ± 14.96 vs. 8.61 ± 13.05; *p* = 0.843). Physical therapy sessions, thumb injuries and boxer’s knuckle were also found to be significantly higher in the boxers group. On the other hand, upper limb surgeries were significantly less in the boxers group. Finally, the size of boxing gloves was associated to the number of finger fractures, thumb injuries and ulnar sided wrist pain in boxers.

**Conclusions:**

Although a controversial sport, boxing appears to have no long-term consequences to the upper limb function, especially regarding hand performance. The size of gloves during heavy bag training was found to be an aggravating factor for hand injuries.

**Supplementary Information:**

The online version contains supplementary material available at 10.1186/s13102-022-00558-3.

## Introduction

Over the years, boxing has become a point of controversy, with increased research focusing on the head injuries, their acute life threatening and chronic consequences (CTE, Dementia Pugilistica) and ways to reduce these [1–7]. However, a big part of boxing injuries consists of upper limb trauma and overuse syndromes with an estimated incidence from 25 to 68% [8–10]. Loosemore et al. [11] explored prospectively the hand and wrist injuries in the Great Britain (2005–2012) and found that finger carpometacarpal instability and “boxer’s knuckle” injuries were significantly more common; also, the rate of these injuries was higher in competition than in training (347 hand injuries/1000 h in competition versus 30 hand injuries/1000 h of punching training) and upper limb injuries incidence was calculated at 55% [10, 11]. Lemme et al. [12] investigated the epidemiology of boxing-related upper-extremity injuries in the United States (2012–2016) and found a mean incidence of 673 injuries per 100,000 person-years, with hand fractures being the most common (132 per 100,000 person-years). The majority of boxers sustaining such injuries were male (84.4%) and between the ages of 20–39 (59.9%) although a 33% injury reduction was observed following rule changes made by sanctioning organizations in 2013 [12]. The purpose of our study was to evaluate the functional status as well as the most common upper limb injuries in Greek boxers and to compare them with military recruits representing the general population. Regarding functional status, the goal was to assess the participants ability and pain levels to perform everyday tasks. Predisposing factors to injury during training or competition were also gathered and associated with quick-DASH and PRWE. Finally we investigate whether glove size, bandage length, percentage of heavy bag drills and number of competition matches influence upper limb functional status by performing dependency analyses between them and quick-DASH and PRWE.

## Material—methods

An observational longitudinal survey was conducted by the Orthopaedic Clinic of Patras University Hospital. Ethical approval to collect medical information and self-administered evaluation forms in healthy individuals was obtained from the Ethical Committee of Patras University Hospital (No: AΠ 231/22-11-2020). Signed consent was also obtained by all responders who agreed to participate. An electronic questionnaire (Additional file 1) was sent to all male members of the Hellenic Boxing Federation. This squad consisted of amateur boxers, some of which were Olympian athletes. The majority of athletes had been training for more than 5 years (65.7%) and spent more than 15 h per month (79.3%) for training, meaning more than three times per week. In addition, 71.2% of boxers had participated at least once to an official boxing match while 7.3% of boxers had participated in more than 50 boxing matches. The electronic questionnaire consisted of three sections: In the first section, demographic data were gathered along with training parameters such as percentage of heavy bag drills and sparring, number of boxing matches, years of training, hours of training per month, length of bandages and the use of head gear. These parameters were analyzed later to recognize predisposing factors for serious injuries or worse functional status of the upper limb. On the second part of the questionnaire, people were asked about the most frequent injuries such as shoulder-elbow-wrist-finger fractures or dislocations, wrist clunking, wrist ganglion, thumb injuries, boxer’s knuckle, concussions, upper limb surgery, number of doctor’s visits, sessions of physical therapy and longest duration of absence from activities (Table 1). The commonest injuries were chosen as found by other studies [11, 12] as well as our own estimations. Photographies of injuries were used for clarification in this section. Finally, the third part included the quick-DASH score and PRWE scale to evaluate the functional status of the upper limb. Analytic descriptions were used throughout the whole questionnaire to assist the participants. The quick-DASH score measured pain levels during everyday activities for the whole upper limb (shoulder, arm, elbow, forearm, wrist, hand) whereas the PRWE score concentrated on the wrist and hand. The same questionnaire was sent to male military recruits; using the Greek Military Ranking tables (2021) we generated a computerized random-numbering system to retrieve 200 young adults (out of ~ 1000) to serve as the control group. Greece has an obligatory military service system for every male and the questionnaire was randomly sent to new military recruits as described above, during their first week of recruitment and before they received any military training. As a result, the above control group is believed to reflect the general population and all conclusions can be generalized. The questionnaire had the same sections except those of training circumstances. For both groups the main exclusion criterion was age > 35 years old and for the non-boxing squad the participation in marital arts at any time in the past.

**Table 1:** Incidence of possible predisposing factors for poor functional status among Boxers and non-Boxers and a comparison among the two groups

Predisposing factors investigated	Non-Boxers (N = 75)	Boxers (N = 62)	*P* value
Weight			**0.037**
< 80 kg	43 (57.33%)	47 (75.81%)	
≥ 80 kg	32 (42.67%)	15 (24.19%)	
No of very serious injuries			0.925
0	37 (49.33%)	32 (51.61%)	
> 0	38 (50.67%)	30 (48.39%)	
No of concussions			0.799
0	64 (85.33%)	51 (82.26%)	
> 0	11 (14.67%)	11 (17.74%)	
Shoulder fracture			1.000
No	71 (94.67%)	58 (93.55%)	
Yes	4 (5.33%)	4 (6.45%)	
Shoulder dislocation			0.752
No	72 (96%)	61 (98.39%)	
Yes	3 (4%)	1 (1.61%)	
Biceps brachii tear or strain			0.359
No	57 (82.61%)	53 (89.83%)	
Yes	12 (17.39%)	6 (10.17%)	
Forearm fracture			0.268
No	66 (90.41%)	59 (96.72%)	
Yes	7 (9.59%)	2 (3.28%)	
Wrist fracture			0.372
No	59 (83.1%)	47 (90.38%)	
Yes	12 (16.9%)	5 (9.62%)	
Fracture in fingers or metacarpals			0.993
No	56 (75.68%)	41 (77.36%)	
Yes	18 (24.32%)	12 (22.64%)	
Wrist sprain			0.617
No	56 (74.67%)	43 (69.35%)	
Yes	19 (25.33%)	19 (30.65%)	
Wrist ganglion			0.344
No	70 (93.33%)	54 (87.1%)	
Yes	5 (6.67%)	8 (12.9%)	
Instability or clunk sounds during wrist movement			0.201
No	33 (44%)	35 (56.45%)	
Yes	42 (56%)	27 (43.55%)	
Boxer’s knuckle			**0.036**
No	67 (89.33%)	46 (74.19%)	
Yes	8 (10.67%)	16 (25.81%)	
Thumb injuries			**< 0.001**
No	64 (85.33%)	32 (51.61%)	
Yes	11 (14.67%)	30 (48.39%)	
Ulnar sided wrist pain			*0.087*
No	69 (93.24%)	51 (82.26%)	
Yes	5 (6.76%)	11 (17.74%)	
Doctor visits			0.427
0	16 (27.12%)	22 (35.48%)	
> 0	43 (72.88%)	40 (64.52%)	
Physical therapy sessions			**0.012**
0	50 (83.33%)	38 (61.29%)	
> 0	10 (16.67%)	24 (38.71%)	
Longest duration of absence from activities			0.151
Days	32 (42.67%)	35 (56.45%)	
Weeks	43 (57.33%)	27 (43.55%)	
Surgery for injury in the upper limbs			**0.005**
No	64 (85.33%)	62 (100%)	
Yes	11 (14.67%)	0 (0%)	
DASH SCORE	15.65 ± 10.25	12.55 ± 8.62	**0.020**
PRWE SCORE	9.25 ± 14.96	8.61 ± 13.05	0.843

Bold indicates statistical importance

### Statistical methods

A set of categorical questionnaire response variables were available for analysis, along with the two scale scores: quick-DASH and PRWE. The categorical variables were described with their count (%) per level, while the scale variables with their mean ± standard deviation. Since the Shapiro Wilk test demonstrated non-normality of the scale scores, hypothesis testing was based on a set of non-parametric tests. Categorical variables were mutually associated with Pearson’s chi squared test of independence. The scale score values were compared regarding levels of the categorical variables with Wilcoxon’s rank sum test or with Kruskal Wallis test, depending on whether the number of levels of the categorical variable was 2 or bigger, respectively. For the visualization of the outcomes, either bar plots or violin plots were utilized, depending on if the association was between categorical variables, or a scale score and a categorical variable. All tests were two-sided, and statistical significance was taken when *p* < 0.05. The overall process of the statistical analysis, processing, and visualization was held with the R statistical language and the RStudio IDE, two well-known open-source products for data analysis.

## Results

**Fig. 1:**
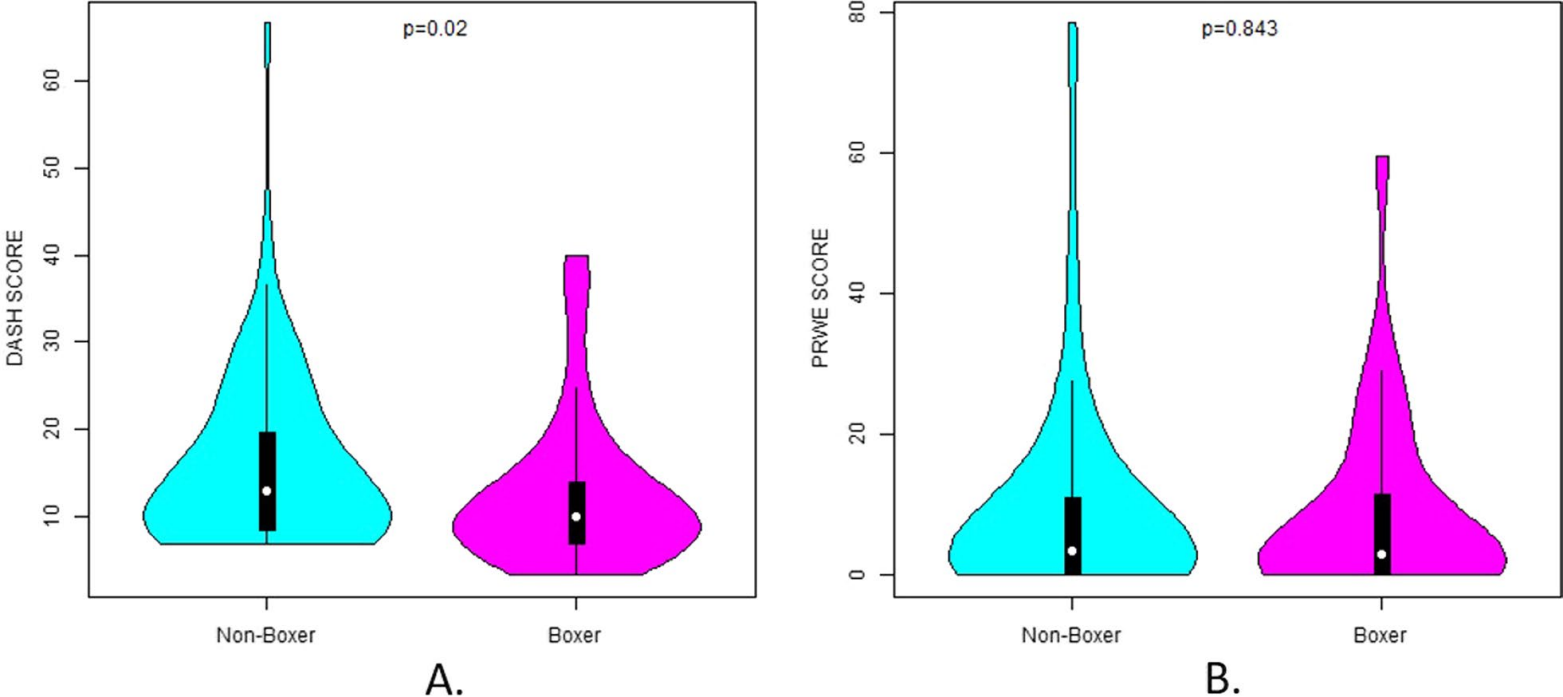
A comparison between boxers and non-boxers in the cohort regarding **A** their DASH score, **B** their PRWE score

A total of 113 boxers and 147 non-boxers answered the questionnaire. After inclusion and exclusion criteria were met, 62 boxers and 75 non boxers were finally compared. The large number of participants excluded is due to the participation of females and males older than 35 years old, whereas for the non-boxers group previous martial arts engagement also led to exclusion. As we did not have a sufficient number of participants on the older than 35 years old and the female gender groups, after power analysis was performed, we decided to exclude these groups. The body mass was found to be significantly higher in the non-boxing population (*p* = 0.037), but this was to be expected, as an athletic population was compared to a non-athletic. As far as the injuries were concerned, only physical therapy sessions (*p* = 0.012), thumb injuries (*p* < 0.001) and boxer’s knuckle (*p* = 0.036) were reported significantly higher among the boxers (Table 1). The number of concussions, shoulder fractures and dislocations, biceps brachii tears, forearm and wrist fractures, wrist sprains, wrist ganglion, wrist instability, ulnar sided wrist pain and doctor visits were not found to differ statistically significant between the boxers and the military recruits. The mean quick-DASH score was found to be significantly lower (better) in the boxers’ (*p* = 0.02), along with the upper limb surgery rates; in contrast, the mean PRWE scale was the same in both groups (Fig. 1).

Apart from these, a dependency analysis was performed in the boxers’ group, between the demographics, the training parameters, and the injuries in relation to the quick-DASH and PRWE scores (Table 2). An association between high quick-DASH score and instability or clunk sounds during wrist movement (*p* = 0.015), finger fractures (*p* = 0.028) and very serious injuries (*p* = 0.044) was found. Long absence from training was associated to both higher quick-DASH and PRWE (*p* < 0.001 and 0.014 respectively). It appears particularly interesting that for both scores, no dependency was found on the weight, the number of injuries, number of boxing matches, glove size, head gear, bandages’ length, heavy bag training and hours of training.

**Table 2:** Association of possible predisposing factors with DASH and PRWE scores in the boxer’s subgroup

Association of possible predisposing factors with DASH and PRWE scores	Count (N)	DASH SCORE	*P* value	PRWE SCORE	*P* value
Weight			*0.089*		0.166
< 80 kg	47	11.12 ± 6.57		7.33 ± 11.66	
≥ 80 kg	15	17.02 ± 12.38		12.63 ± 16.49	
Years of training			0.33		0.761
≤ 5 years	27	13.62 ± 9.45		9.3 ± 14.01	
> 5 years	35	11.72 ± 7.95		8.09 ± 12.44	
Hours of training per month			0.839		0.987
≤ 15 h	16	12.34 ± 8.75		8.12 ± 10.07	
> 15 h	46	12.62 ± 8.67		8.78 ± 14.03	
Percentage of physical training			0.631		0.252
< 60%	35	13.54 ± 9.52		7.47 ± 12.66	
≥ 60%	27	11.26 ± 7.25		10.09 ± 13.63	
Percentage of heavy bag drills			0.296		0.392
< 40%	30	12.21 ± 9.17		6.67 ± 9.58	
≥ 40%	32	12.87 ± 8.19		10.44 ± 15.56	
Percentage of sparring			0.877		0.518
< 60%	39	12.19 ± 8.14		7.67 ± 12.2	
≥ 60%	23	13.16 ± 9.53		10.22 ± 14.51	
Head gear usage			0.691		0.801
No	30	12.51 ± 8.14		8.83 ± 13.49	
Yes	32	12.58 ± 9.17		8.41 ± 12.83	
Length of hand bandages			0.443		0.247
< 4 m	37	12.1 ± 8.66		7.69 ± 13.14	
≥ 4 m	25	13.21 ± 8.69		9.98 ± 13.05	
Size of gloves during heavy bag drills			0.808		0.426
Leather/Bandage	5	19 ± 14.63		4.1 ± 8.62	
< 14 oz	37	11.45 ± 6.72		9.96 ± 15.28	
≥ 14 oz	20	12.96 ± 9.71		7.25 ± 8.83	
Stretching before training			0.329		0.616
No	3	8.07 ± 1.72		2.83 ± 3.33	
Yes	59	12.78 ± 8.77		8.91 ± 13.3	
Participation in boxing matches			0.161		0.543
No	12	16.52 ± 12.23		9 ± 13.09	
Yes	50	11.59 ± 7.35		8.52 ± 13.17	
If yes. how many boxing matches			0.33		0.152
≤ 25 matches	40	11.76 ± 7.75		8.56 ± 13.89	
> 25 matches	6	13.23 ± 6.18		12.92 ± 11.27	
Duration of absence from systematic training			0.224		0.239
< 1 year	40	13.38 ± 9.09		9.59 ± 14.42	
≥ 1 year	22	11.03 ± 7.65		6.84 ± 10.16	
No of very serious injuries			**0.044**		0.551
0	32	10.04 ± 4.82		7.66 ± 12.36	
> 0	30	15.23 ± 10.81		9.63 ± 13.88	
No of concussions			0.231		0.441
0	51	12.33 ± 8.81		9.74 ± 14.05	
> 0	11	13.55 ± 7.99		3.41 ± 3.88	
Shoulder fracture			0.348		0.737
No	58	12.88 ± 8.81		8.55 ± 13.27	
Yes	4	7.7 ± 0.4		9.5 ± 10.79	
Shoulder dislocation			0.102		0.955
No	61	12.7 ± 8.6		8.71 ± 13.13	
Yes	1	3.2 ± NA		2.5 ± NA	
Biceps brachii tear or strain			*0.092*		0.572
No	53	11.65 ± 8.07		8.42 ± 13.56	
Yes	6	19.15 ± 12.52		6.92 ± 7.36	
Forearm fracture			0.951		0.62
No	59	12.38 ± 8.18		8.63 ± 13.34	
Yes	2	19.5 ± 23.05		5 ± 2.12	
Wrist fracture			0.443		*0.077*
No	47	12.73 ± 8.74		7.69 ± 12.05	
Yes	5	16.2 ± 12.47		11.4 ± 10.1	
Fracture in fingers or metacarpals			**0.028**		0.168
No	41	11.13 ± 7.27		7.51 ± 11.98	
Yes	12	18.98 ± 11.64		11.33 ± 11.7	
Wrist sprain			*0.072*		*0.062*
No	43	11.22 ± 7.61		6.6 ± 10.91	
Yes	19	15.56 ± 10.13		13.16 ± 16.36	
Wrist ganglion			0.767		1
No	54	12.25 ± 8.23		9.01 ± 13.6	
Yes	8	14.56 ± 11.35		5.94 ± 8.58	
Instability or clunk sounds during wrist movement			**0.015**		*0.069*
No	35	9.63 ± 4.01		7.24 ± 12.42	
Yes	27	16.33 ± 11.26		10.39 ± 13.86	
Boxer’s knuckle			0.436		0.485
No	46	12.6 ± 9.28		8.09 ± 12.04	
Yes	16	12.41 ± 6.62		10.12 ± 15.94	
Thumb injuries			0.57		0.62
No	32	11.63 ± 5.48		8.09 ± 13.34	
Yes	30	13.52 ± 11.05		9.17 ± 12.93	
Ulnar sided wrist pain			0.178		0.114
No	51	11.44 ± 7.28		6.99 ± 10.89	
Yes	11	17.71 ± 12.35		16.14 ± 19.23	
Doctor visits			0.17		0.736
0	22	10.25 ± 5.55		8.16 ± 13.86	
> 0	40	13.81 ± 9.74		8.86 ± 12.75	
Physical therapy sessions			0.625		0.123
0	38	11.5 ± 6.85		7.22 ± 12.21	
> 0	24	14.2 ± 10.8		10.81 ± 14.26	
Longest duration of absence from activities			**< 0.001**		**0.014**
Days	35	9.29 ± 5.77		5.73 ± 11.28	
Weeks	27	16.78 ± 9.89		12.35 ± 14.4	

Bold indicates statistical importance

A separate analysis was performed for concussions. The number of concussions were classified as 0 or more but no relation was found between years of training, percentage of sparring, head gear, gloves’ size, and boxing matches. Finally, percentage of heavy bag drills, size of gloves, length of bandages and number of boxing matches were correlated to the most common injuries. The results were statistically significant only when comparing thumb injuries and finger fractures to the size of gloves during heavy bag drills (*p* = 0.022 and *p* = 0.032 respectively), but conversely we observed that finger fractures and ulnar sided wrist pain was correlated to higher size of boxing gloves.

## Discussion

### Overall functional status

Perhaps the most important conclusions of this study, is the absence of difference on the PRWE score between the boxers and the general population, and the lower (better) DASH score of the boxers’ group (Fig. 1). This shows that boxing reserves no serious long-term consequences regarding the functional status of the hands and arms of the average athlete, at least for the age groups compared. Consequently, it would be really interesting to examine this in older boxers (where perhaps the decreased musculature will give different results) or in professional boxers, as already a difference between competition and training is presented in research [11]. Apart from that, no difference was found in the prevalence of concussions, shoulder fractures, shoulder dislocations, biceps brachii strains, forearm fractures, wrist fractures, wrist sprains, fractures in metacarpals, wrist ganglion, carpal tunnel syndrome, wrist instability and ulnar sided wrist pain. However, physical therapy sessions (*p* = 0.012), thumb injuries (*p* < 0.001) and boxer’s knuckle (*p* = 0.036) occurred significantly more often in boxers, whereas the boxers faced fewer upper limb surgeries (*p* = 0.005). This comes in partial agreement to one of the largest studies conducted about hand and wrist injuries in boxing, as Loosemore et al. [10] found in 2016 that the 3 most common injuries were carpometacarpal (CMC) instability (21.6%), boxer’s knuckle (15.8%) and skier’s thumb (14.6%). Interestingly, wrist clunking (possibly correlated with carpometacarpal instability) was also correlated with higher DASH score in our study (*p* = 0.015). On the other hand, in our study we met a lower prevalence of upper limb surgeries. The explanation we could offer, speculatively, is that the increased musculature of the boxers compensates for the anatomical impairment of an injury and the loss of stability caused, thereafter decreasing the need for surgery. Lemme et al. [12] found the hand fractures to be one of the most common injuries among boxers, but this was a study conducted in emergency departments. Perhaps the severity (as found by the current study) of the hand fractures might bring those boxers to the emergency department often, but the real incidence is not so high, as neither our nor Loosemore et al. [11] could verify this finding. Finally, the high incidence of physical therapy sessions only among boxers, as highlighted by our study, could be an indicator of underreported injuries in the boxing population, as noted in our Limitations section.

### Boxer’s knuckle and skier’s thumb (Fig. 2)

Boxer’s knuckle is more common among boxers according to our and numerous other studies [13, 14]. The severity of the injury can extend from mild capsular injury to frank dislocation of the extensor tendon. Diagnosis is usually clinical, but dynamic imaging is of high value, such as dynamic ultrasonography [15]. Conservative treatments such as splinting in extension, rest and medication can be applied successfully for mild injuries, but surgical treatment, with excellent return to play rates, should be preferred when there is a tendon dislocation as these do not respond well to conservative treatment, possibly due to fistula formation between the subcutaneous tissue and the capsule, and the development of chondromalacia/osteoarthritis if left untreated [16–20]. Skier’s thumb and thumb fractures although common among boxers, as verified by our and other studies, are not nearly as well documented as boxer’s knuckle, carpometacarpal bossing, or boxer’s fractures in the boxers’ population [21]. Undisplaced fractures or partial ulnar collateral ligament tears are treated with splinting in mild flexion for 4–6 weeks, whereas chronic tears, unstable displaced fractures, Stener’s lesions, acute unstable tears and volar plate injuries are indicated for surgical management including direct repair, reconstruction, arthrodesis, or arthroplasty [22, 23].

**Fig. 2:**
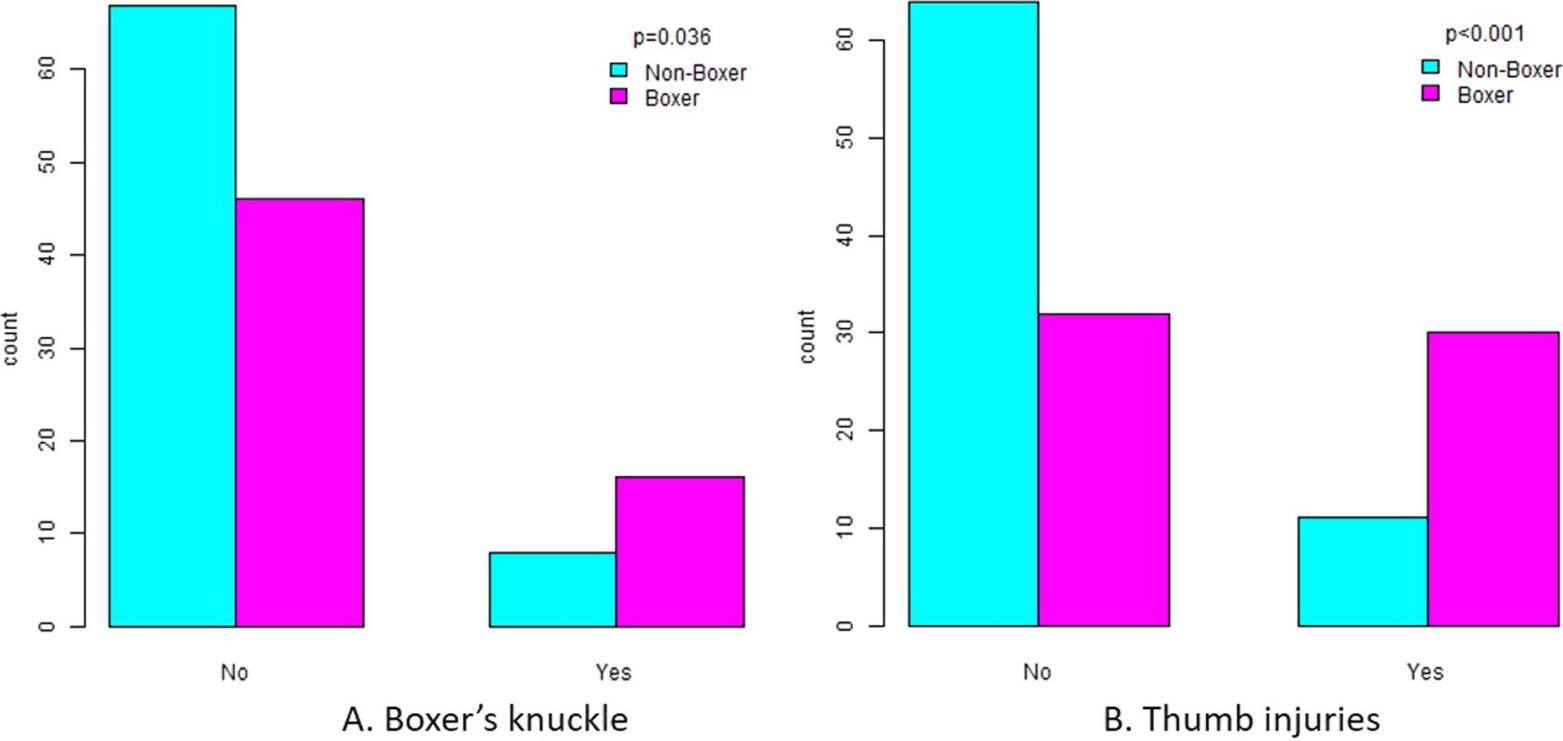
A comparison between boxers and non-boxers in the cohort regarding **A** their boxer's knuckles, **B** their thumb injuries

### Glove size

**Fig. 3:**
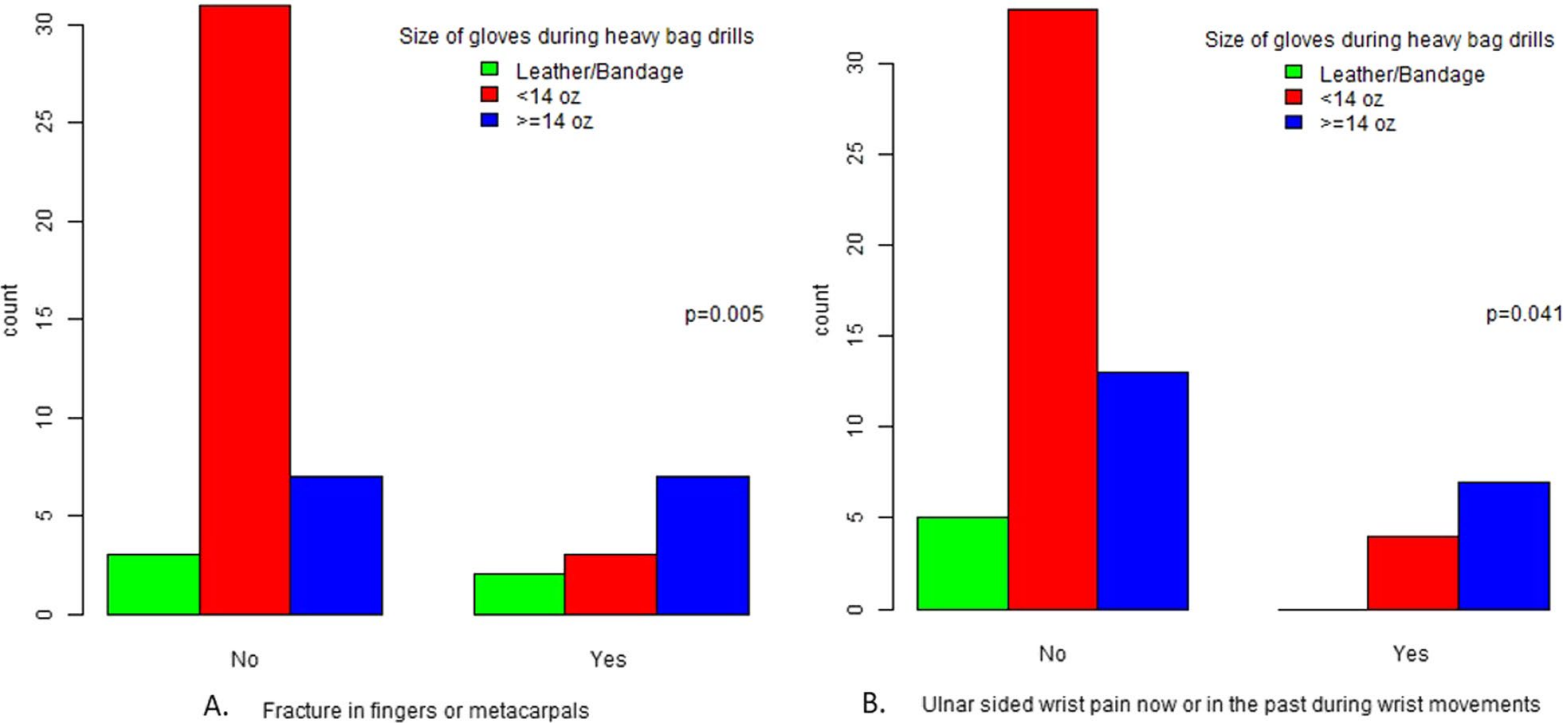
The association between the gloves' size and **A** finger/metacarpal fractures, **B** ulnar sided wrist pain, in the boxers' sub cohort

The current study found strong association between size of gloves and finger fractures (*p* = 0.005) or ulnar sided wrist pain (*p* = 0.041), as the higher the size of gloves, the higher the incidence of these injuries (Fig. 3). In 2013, the AIBA (as of last year rebranded as International Boxing Association to signal a wide set of reforms) changed the regulations, removing the headgear, increasing the glove size and changing the scoring system [12]. This resulted to a change in the fighting style, as more long-range jabs were used instead of the hook and cross, which put the shoulder in the vulnerable position of external rotation and abduction and produce more force [24], while the bigger gloves supposedly provided better hand protection [25]. At the same time, a study conducted from 2012 to 2016 in US, found a lower rate of reported injuries from boxing after the change of rules in 2013 [12]. The traditional training technique would be to use bigger gloves during punching bag sessions. That is supported by lab research, as Lee & McGill e al. found that thicker gloves with less stiff padding present lower peak forces and lower loading rates, as expected [26]. So the advantages assumed of this practice are injury prevention through the heavier padding and higher hand speed during competition, where the gloves are lighter. However, bigger gloves could mean less stability and more rollover during punching, which could lead to more fractures and ligamentous injuries, as observed from our study. Or as Lee & McGill noted, padding deterioration on heavy gloves can have the opposite effect, as material failure can show higher peak forces and faster loading rates [26]. Another more plausible explanation could be that these injuries pre-existed and led the athletes to bigger glove size, as some pre-existing injuries could also be associated with worse functional status. Unfortunately, some of the above is speculation, and is provided after discussion with coaches and athletes. We report it to provide consideration for future research on the effect of glove size in boxing.

### Other common injuries

Another well documented injury, the boxer’s fracture, is a fracture of the 4^th^ or 5^th^ metacarpal. However, the literature suggests that this injury is not met during boxing rather during bouts with bare knuckles [19], a finding confirmed by our study as we did not record a statistically significant higher incidence of boxer’s fracture among boxers. Finally, carpometacarpal (CMC) bossing is another common injury among boxers. It is well documented that there is little degree of movement in the 2nd and 3rd carpometacarpals (1° and 3° respectively) and more for the 4th and 5th carpometacarpals (8°–15° and 15°–40° respectively) [27–29]. This is one of the reasons why boxers hit the bag with their index and middle finger [30], when practicing correct punching technique. Despite the low degree of mobility in the 2nd and 3rd CMC, repetitive impact can cause a flexion downward force, with secondary dorsal flexion at the CMC joint. The first choice of treatment includes conservative management with immobilization, but recurrent injuries can result in bony hypertrophy, degenerative changes, and joint subluxation, requiring CMC arthrodesis [20, 25]. Other upper limb boxing injuries reported sparingly in the literature include avulsion fracture of the ECRB insertion in trapezium, scapula fractures, internal impingement of the coronoid and olecranon processes, teres major tendon tears and flexor carpal radialis avulsion [31–35].

### Limitations

Unfortunately, there are multiple limitations to this study, which we should recognize. First of all, it is a retrospective study and the data collected were self-reported by the participants. No medical diagnosis was done for the conditions studied. As mentioned by Finlay et al. and Altaribba-Bartes et al. self reported data in boxing might not be always accurate [36, 37]. This could be attributed to the criticizing boxing is receiving for being dangerous (coaches want to highlight the numerous benefits of boxing so that more people join the sport) as well as due to the boxer’s mentality to fight through the injury especially before a bout [36, 37]. What is more, there is the side of boxing that is associated with underground activities and officially recording of any kind is not welcome by some clubs. The groups were only helped through photos and descriptions. Apart from that, no differences between injuries suffered during competition or training could be studied. Finally, there was not sufficient sample size to study the functional status of veteran (> 35 years-old) or female boxers, which would be really interesting.

## Conclusions

Overall, multiple studies agree that upper limb injuries constitute a major part of boxing trauma, with the danger being much greater during competition, but without any serious long-term consequences for the amateur boxers as highlighted here. In our study, finger fractures and wrist clunking, which could indicate carpometacarpal instability, were linked to poorer functional status. For this reason, more research needs to be held in terms of identifying preventing factors for injuries such as carpometacarpal instability, boxer’s knuckle, finger fractures and skier’s thumb, as they present a high incidence or poor prognosis. For the time being, the traditional protective methods should be employed as described by the coaches, such as long bandages even coupled with foam, taping around the wrist, at least two weeks of absence after serious wrist sprains and correct punching technique (punching with the wrist slightly flexed, so that the metacarpals and the radius are in line, and making contact with the index and middle finger). The effect of strength and conditioning training towards injury prevention at the wrist should also be studied, considering that benefits on injury reduction have been observed in various sports [38, 39] and the implementation on boxing populations is low, especially among Senior Development Boxers (SDB) [36]. Further studies should be conducted regarding the functional status of veteran boxers, female boxers, as well as the association of glove size and injuries. Finally, the use of the Takei handheld dynamometer has been proven reliable and valid in boxers’ hand injuries and could assist in the direction of underreported injuries among this population [40].

## Supplementary Information

**Additional file 1.** Functional Assesment of Boxers in Greece.

## Data Availability

The datasets used and/or analyzed during the current study are available from the corresponding author on reasonable request.

## Abbreviations

PRWE: Patient Rated Wrist Evaluation scale
Quick-DASH: Quick Disabilities of Arm, Shoulder and Hand score
CMC: Carpometacarpal instability
IBA: International Boxing Association
ECRB: Extensor Carpi Radialis Brevis
