# Supplementary material for: Functional performance of the upper limb and the most common boxing-related injuries in male boxers: a retrospective, observational, comparative study with non-boxing population
Source: BMC Sports Sci Med Rehabil. 2022 Sep 1;14:162. doi: 10.1186/s13102-022-00558-3 (PMC9434859; doi:10.1186/s13102-022-00558-3)
Supplement: Supplementary file 1 — Additional file 1. Functional Assesment of Boxers in Greece. [file 13102_2022_558_MOESM1_ESM.pdf]

Functional Assesment of Boxers in Greece

The following questionnaire is part of a retrospective study. Aim of our study is to assess the functional status of upper limb and wrist in Boxers and identify risk factors and suggest safety measures during training. Approximately 10 minutes will be needed.
Thank you for your time !

* Απαιτείται

1. Sex *

   Να επισημαίνεται μόνο μία έλλειψη.

   - Male
   - Female

2. Age *

   Να επισημαίνεται μόνο μία έλλειψη.

   - <18 years old
   - 18-35 years old
   - >35 years old

3. Profession (optionally)

   ____

4. How many kgs were you during training? *

   Να επισημαίνεται μόνο μία έλλειψη.

   - 40-60 kg
   - 60-80 kg
   - 80-95 kg
   - >95 kg

5. Total years of boxing *

Να επισημαίνεται μόνο μία έλλειψη.

- <1 years
- 1-5 years
- >5 years

6. Hours of training per month *

Να επισημαίνεται μόνο μία έλλειψη.

- <4 hours
- 5-15 hours (1-3 times per week)
- >15 hours(>3 times per week)

7. From which physical conditioning training constituted: *

Να επισημαίνεται μόνο μία έλλειψη.

- 0%
- 20%
- 40%
- 60%
- 80%
- 100%

8. From which heavy bag training constituted: *

Να επισημαίνεται μόνο μία έλλειψη.

- 0%
- 20%
- 40%
- 60%
- 80%
- 100%

9. From which sparring training constituted: *

Να επισημαίνεται μόνο μία έλλειψη.

- 0%
- 20%
- 40%
- 60%
- 80%
- 100%

10. Did you use headgear *

Να επισημαίνεται μόνο μία έλλειψη.

- No
- Seldom
- Often
- Always

11. How many meters bandages did you use *

Να επισημαίνεται μόνο μία έλλειψη.

- 2-2,5 meters
- 3-3,5 meters
- 4-4,5 meters
- 5 meters

12. What size of gloves did you use during heavy bag training *

Να επισημαίνεται μόνο μία έλλειψη.

- Leather/bandages
- 8 oz
- 10 oz
- 12 oz
- 14 oz
- 16 oz

13. Did you stretch before training? *

Να επισημαίνεται μόνο μία έλλειψη.

- No
- Sometimes
- Always

14. Participation in boxing matches *

Να επισημαίνεται μόνο μία έλλειψη.

- Yes
- No

15. If yes, in how many matches have you participated?

____

16. How long are you currently absent from systematic training? *

Να επισημαίνεται μόνο μία έλλειψη.

- <1 year
- 1-3 years
- 3-7 years
- >7 years

History of Injuries

The following questions are regarding serious injuries caused during boxing training or matches.

17. How many really serious injuries do you remember having during training or match? *

Να επισημαίνεται μόνο μία έλλειψη.

- None
- 1
- 2-4
- >5

18. Have you ever had a concussion? *

Να επισημαίνεται μόνο μία έλλειψη.

- No
- 1-2 times
- 3-10 times
- >10 times

19. Have you ever had a shoulder fracture? *

Να επισημαίνεται μόνο μία έλλειψη.

- Yes
- No

20. Have you ever had a shoulder dislocation? *

Να επισημαίνεται μόνο μία έλλειψη.

- No
- 1
- >=2

21. Have you ever had a biceps strain or rupture? *

Να επισημαίνεται μόνο μία έλλειψη.

- Yes
- No
- Maybe

22. Have you ever had a forearm fracture? *

Να επισημαίνεται μόνο μία έλλειψη.

- Yes
- No
- Maybe

23. Have you ever had a wrist fracture? *

Να επισημαίνεται μόνο μία έλλειψη.

- Yes
- No
- Maybe

24. Have you ever had a finger or metacarpal fracture? *

Να επισημαίνεται μόνο μία έλλειψη.

- Yes
- No
- Maybe

25. Have you ever had a wrist sprain? *

Να επισημαίνεται μόνο μία έλλειψη.

- Yes
- No
- Maybe

26. Have you ever had a ganglion? *

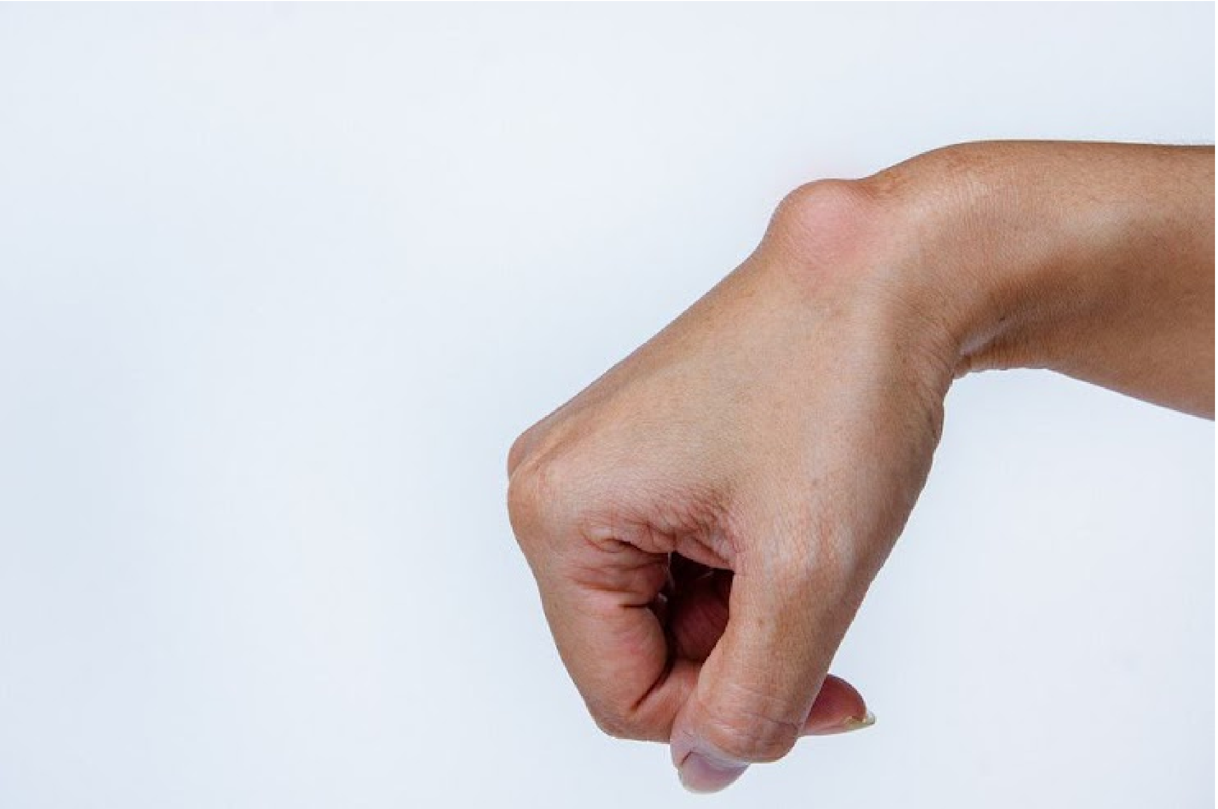

Να επισημαίνεται μόνο μία έλλειψη.

- Yes
- No

27. Do you present instability or crepitus during wrist movement? *

Να επισημαίνεται μόνο μία έλλειψη.

- Yes
- No
- Maybe

28. Do you present the deformity shown in the picture? *

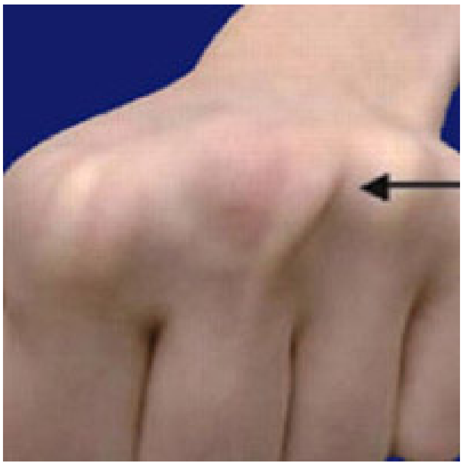

Να επισημαίνεται μόνο μία έλλειψη.

- Yes
- No

29. Did you have any thumb injuries? *

Να επισημαίνεται μόνο μία έλλειψη.

- Yes
- No
- Maybe

30. Do you present ulnar sided wrist pain during movements, as shown in the photo? *

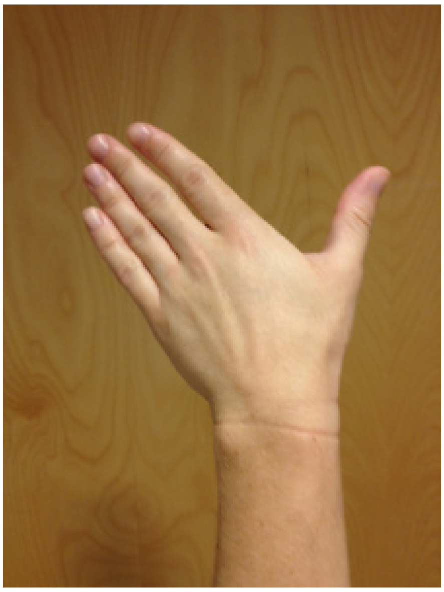

Να επισημαίνεται μόνο μία έλλειψη.

- [ ] A lot
- [ ] Moderately
- [ ] Minimally
- [ ] Not at all

31. How many times have you visited a doctor for injury during boxing? *

Να επισημαίνεται μόνο μία έλλειψη.

- [ ] None
- [ ] 1
- [ ] 2-3
- [ ] >=4

32. How many physical therapies have you had for injuries related to boxing? *

Να επισημαίνεται μόνο μία έλλειψη.

- 0
- 1
- 2-3
- >=4

33. What is the longest duration you have abstained from boxing due to injury? *

Να επισημαίνεται μόνο μία έλλειψη.

- Few days
- Few weeks
- Few months
- >6 months

34. If you have sustained an upper limb fracture, how did you manage it? *

Να επισημαίνεται μόνο μία έλλειψη.

- Nothing
- Bandaging
- Splinting
- Plaster
- Surgery

35. Have you had surgery for upper limb injury? *

Να επισημαίνεται μόνο μία έλλειψη.

- Yes
- No

Upper Limb Functional Assesment - Quick DASH

36. Please rate your ability to do the following activities in the last week: *

Να επισημαίνεται μόνο μία έλλειψη ανά σειρά.

	NO DIFFICULTY	MILD DIFFICULTY	MODERATE DIFFICULTY	SEVERE DIFFICULTY	UNABLE
**Open a tight or new jar**	⬭	⬭	⬭	⬭	⬭
**Do heavy household chores (e.g. wash walls, wash floors)**	⬭	⬭	⬭	⬭	⬭
**Carry a shopping bag or briefcase**	⬭	⬭	⬭	⬭	⬭
**Wash your back**	⬭	⬭	⬭	⬭	⬭
**Use a knife to cut food**	⬭	⬭	⬭	⬭	⬭
**Recreational activities in which you take some force or impact through your arm, shoulder or hand (e.g. golf, hammering, tennis, etc.)**	⬭	⬭	⬭	⬭	⬭
**During the past week, were you limited in your work or other regular daily activities as a result of your arm, shoulder or hand problem?**	⬭	⬭	⬭	⬭	⬭

37. *

Να επισημαίνεται μόνο μία έλλειψη ανά σειρά.

	NOT AT ALL	MILD DIFFICULTY	MODERATE DIFFICULTY	SEVERE DIFFICULTY	UNABLE
**During the past week, to what extent has your arm, shoulder or hand problem interfered with your normal social activities with family, friends, neighbours or groups?**					

38. *

Να επισημαίνεται μόνο μία έλλειψη ανά σειρά.

	NOT LIMITED AT ALL	SLIGHTLY LIMITED	MODERATELY LIMITED	VERY LIMITED	UNABLE
**During the past week, were you limited in your work or other regular daily activities as a result of your arm, shoulder or hand problem?**					

39. Please rate the severity of the following symptoms in the last week: *

Να επισημαίνεται μόνο μία έλλειψη ανά σειρά.

	NONE	MILD	MODERATE	SEVERE	EXTREME
**Arm, shoulder or hand pain**	⬭	⬭	⬭	⬭	⬭
**Tingling (pins and needles) in your arm, shoulder or hand**	⬭	⬭	⬭	⬭	⬭

40. *

Να επισημαίνεται μόνο μία έλλειψη ανά σειρά.

	NO DIFFICULTY	MILD DIFFICULTY	MODERATE DIFFICULTY	SEVERE DIFFICULTY	SO MUCH DIFFICULTY I CAN'T SLEEP
**During the past week, how much difficulty have you had sleeping because of the pain in your arm, shoulder or hand?**	⬭	⬭	⬭	⬭	⬭

41. WORK MODULE (OPTIONAL) - The following questions ask about the impact of your arm, shoulder or hand problem on your ability to work (including homemaking if that is your main work role). Please indicate what your job/work is:

____

42. Please circle the number that best describes your physical ability in the past week. Did you have any difficulty: *

Να επισημαίνεται μόνο μία έλλειψη ανά σειρά.

	NO DIFFICULTY	MILD DIFFICULTY	MODERATE DIFFICULTY	SEVERE DIFFICULTY	UNABLE
**using your usual technique for your work?**	⬭	⬭	⬭	⬭	⬭
**doing your usual work because of arm, shoulder, or hand pain?**	⬭	⬭	⬭	⬭	⬭
**doing your work as well as you would like?**	⬭	⬭	⬭	⬭	⬭
**spending your usual amount of time doing your work?**	⬭	⬭	⬭	⬭	⬭

43. SPORTS/PERFORMING ARTS MODULE (OPTIONAL) - The following questions relate to the impact of your arm, shoulder or hand problem on playing your musical instrument or sport or both. If you play more than one sport or instrument (or play both), please answer with respect to that activity which is most important to you. Please indicate the sport or instrument which is most important to you:

____

44. Please circle the number that best describes your physical ability in the past week. Did you have any difficulty: *

Να επισημαίνεται μόνο μία έλλειψη ανά σειρά.

	NO DIFFICULTY	MILD DIFFICULTY	MODERATE DIFFICULTY	SEVERE DIFFICULTY	UNABLE
**using your usual technique for playing your instrument or sport?**					
**. playing your musical instrument or sport because of arm, shoulder or hand pain?**					
**playing your musical instrument or sport as well as you would like?**					
**spending your usual amount of time practising or playing your instrument or sport?**					

Patient Rated Wrist Evaluation

Rate the average amount of pain/difficulty you have had in your wrist over
the past week by circling the number from 0 (no pain or difficulty) to 10 (the
worst pain you have ever experienced or you could not do the task).

45. PAIN *

Να επισημαίνεται μόνο μία έλλειψη ανά σειρά.

	0	1	2	3	4	5	6	7
**At rest**								
**When doing a task with repeat wrist movement**								
**When lifting a heavy object**								
**When it is at its worst**								
**How often do you have pain?**								

46. FUNCTION - SPECIFIC ACTIVITIES: *

Να επισημαίνεται μόνο μία έλλειψη ανά σειρά.

	0	1	2	3	4	5	6	7
**Turn door knob using my affected hand**	◯	◯	◯	◯	◯	◯	◯	◯
**Cut meat using knife in my affected hand**	◯	◯	◯	◯	◯	◯	◯	◯
**Fasten buttons on my shirt**	◯	◯	◯	◯	◯	◯	◯	◯
**Use my affected hand to push up from a chair**	◯	◯	◯	◯	◯	◯	◯	◯
**Carry a 10-lb object with my affected hand**	◯	◯	◯	◯	◯	◯	◯	◯
**Use bathroom tissue with my affected hand**	◯	◯	◯	◯	◯	◯	◯	◯

47. FUNCTIONAL - USUAL ACTIVITIES *

Να επισημαίνεται μόνο μία έλλειψη ανά σειρά.

	0	1	2	3	4	5	6	7
**Personal care activities (dressing, washing)**								
**Household work (cleaning)**								
**Work (your job or everyday work)**								
**Recreational activities**								

Αυτό το περιεχόμενο δεν έχει δημιουργηθεί και δεν έχει εγκριθεί από την Google.

Google Φόρμες
